# Advances and applications of CRISPR/Cas-mediated interference in *Escherichia coli*

**DOI:** 10.1016/j.engmic.2023.100123

**Published:** 2023-11-02

**Authors:** Xiaohui Lim, Congqiang Zhang, Xixian Chen

**Affiliations:** Singapore Institute of Food and Biotechnology Innovation (SIFBI), Agency for Science Technology and Research (A∗STAR), 31 Biopolis Way, Level 6, Nanos Building, Singapore 138669, Singapore

**Keywords:** CRISPRi, Multiplex, Metabolic engineering, CRISPR/dCas9, Retroactivity

## Abstract

The bacterium *Escherichia coli* (*E. coli*) is one of the most widely used chassis microbes employed for the biosynthesis of numerous valuable chemical compounds. In the past decade, the metabolic engineering of *E. coli* has undergone significant advances, although further productivity improvements will require extensive genome modification, multi-dimensional regulation, and multiple metabolic-pathway coordination. In this context, clustered regularly interspaced short palindromic repeats (CRISPR), along with CRISPR-associated protein (Cas) and its inactive variant (dCas), have emerged as notable recombination and transcriptional regulation tools that are particularly useful for multiplex metabolic engineering in *E. coli*. In this review, we briefly describe the CRISPR/Cas9 technology in *E. coli*, and then summarize the recent advances in CRISPR/dCas9 interference (CRISPRi) systems in *E. coli*, particularly the strategies designed to effectively regulate gene repression and overcome retroactivity during multiplexing. Moreover, we discuss recent applications of the CRISPRi system for enhancing metabolite production in *E. coli*, and finally highlight the major challenges and future perspectives of this technology.

## Graphical abstract

**Figure:**
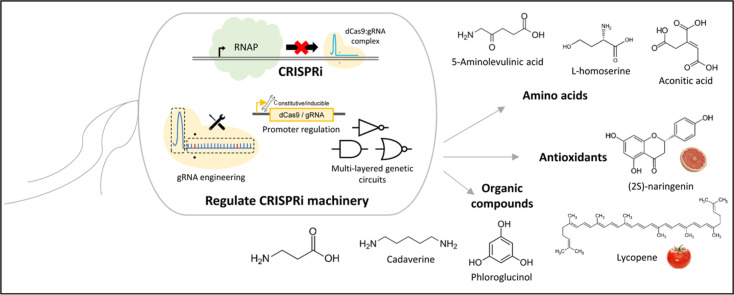

## 1 Introduction to CRISPR/Cas9 technology

**Fig. 1:**
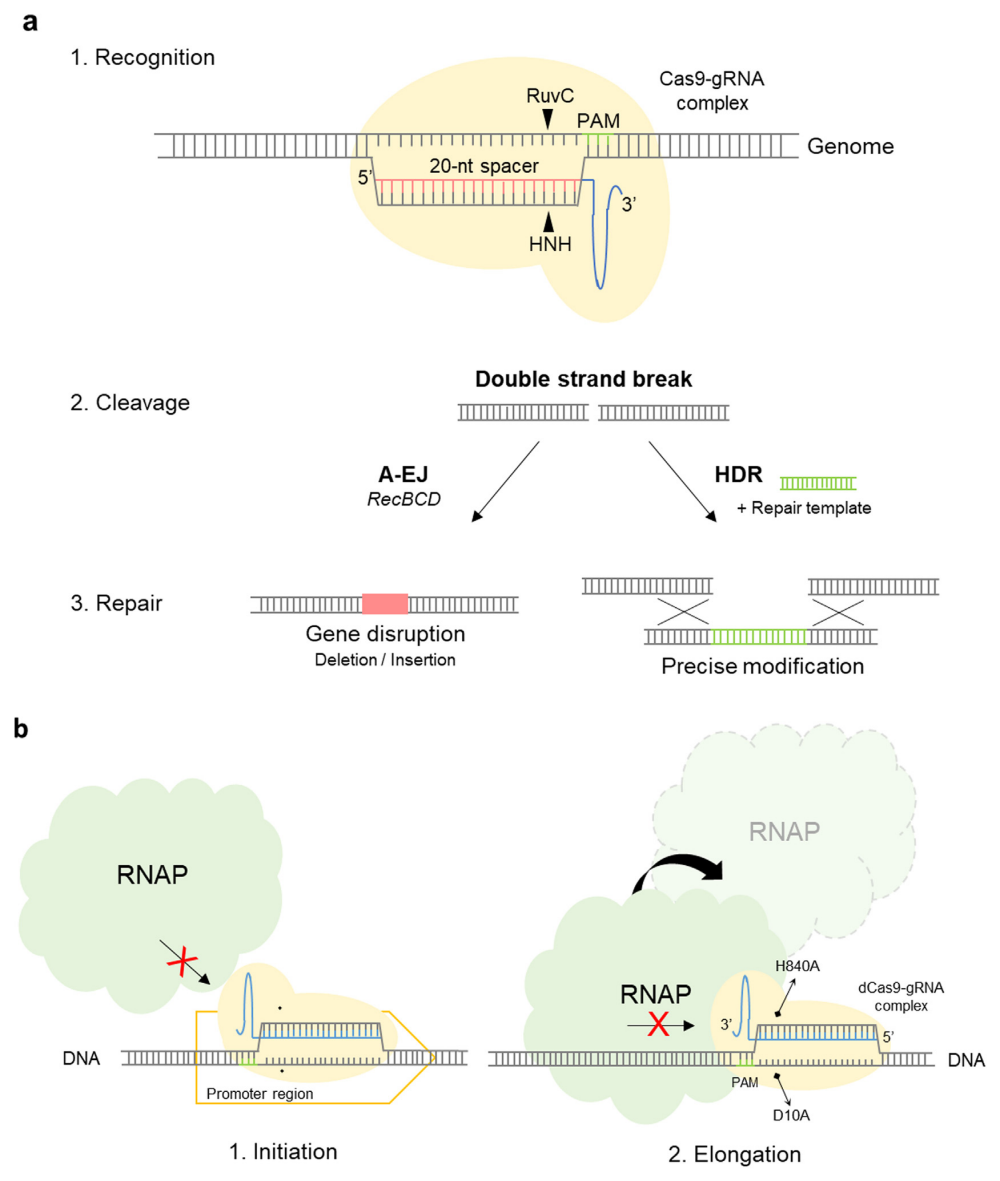
Schematic representation of the CRISPR and CRISPRi machinery **a.** A schematic representation of CRISPR/Cas9 machinery in three steps. 1. Recognition: The Cas9 forms a complex with the gRNA which then directs the Cas9-gRNA complex to the target binding site programmed by the 20-nt guide sequence. 2. Cleavage: The Cas9 nuclease cleaves target DNA, introducing a double stranded break. 3. Repair: DNA repair is then carried out by alternative end-joining (A-EJ) or homology-directed repair (HDR) **b.** A schematic representation of CRISPR/dCas9 machinery. The dCas9 forms a complex with the gRNA which then directs the dCas9-gRNA complex to the target binding site programmed by the 20-nt guide sequence. Thereafter, the dCas9-gRNA complex will block 1. the binding of RNA polymerase interfering with the gene transcription initiation or 2. the progression of the RNA polymerase interfering with the gene transcription elongation.

Since the first production of recombinant human insulin [1], the bacterium *E. coli* has become an extensively used industrial host for recombinant protein production [2,3], DNA replication [4] and chemical synthesis [5]. Its well-characterized genome, and the wide availability of genetic modification tools and genome-scale models [6] facilitate the ready manipulation of this bacterium in multiple applications. Breakthrough titers and yields have been achieved using *E. coli* for small-molecule production from diverse carbon sources [7], [8], [9], [10]. Prior to the discovery of the clustered regularly interspaced short palindromic repeats/CRISPR-associated protein (CRIPSR/Cas) system, modification of the *E. coli* genome was mainly achieved using transposons, phage, or the λ Red recombinase system [11]. However, the transposon and phage approaches lack the flexibility for selecting an integration site, whereas application of the λ Red recombinase system often requires an antibiotic selection marker to enhance recombination efficiency, which leaves a scar within the genome, and necessitates a second round of recombination to remove the selection marker [12,13]. An alternative approach is targeted genome editing mediated by programmable nucleases, such as zinc finger nucleases [14,15] and transcription activator-like effector nucleases [16,17]. However, these methods tend to be limited by the tedious process of redesigning and reengineering a new set of proteins to target different sites, thereby presenting considerable challenges for efficient multiplexing. The recent discovery of the CRISPR/Cas system has revolutionized the biotechnological world owing to its simplicity, programmability, and versatility for diverse applications. The CRISPR/Cas technology offers flexibility, scarless genome modification, precise base editing, ease of multiplexing, and a significant enhancement of recombination efficiency in *E. coli* [18]. The CRISPR/Cas system was initially discovered in the bacterial immune system [19], [20], [21], [22] and has subsequently been found to be present in 45 % of bacteria and 84 % of archaea [20,[22], [23], [24], [25]]. The system generally consists of a CRISPR array and Cas proteins. The CRISPR array comprises a series of short repeated sequences separated by unique spacer sequences [20,26]. The spacer sequences are often foreign viral DNAs derived from previously unsuccessful infections. Cas nucleases can generally be classified into two classes [23,[27], [28], [29], [30], [31], [32], [33], [34], [35]]. Class 1 consists of multi-domain Cas proteins, namely, types I, III, and IV, and their subtypes, whereas Class 2 consists of a single Cas protein effector and types II, V, and VI, harboring Cas9, Cas12, and Cas13 nucleases [23,31,32]. There are currently approximately 180 Cas proteins reviewed in the UniProt database, of which 24 are class 2 Cas nucleases. Among these, Cas9 derived from *Streptococcus pyogenes* (SpyCas9) is the most well characterized and widely used. The Cas9 enzyme comprises two nuclease domains, the HNH and RuvC catalytic domains, which catalyze the cleavage of double-stranded DNA (Fig. 1a) [36], [37], [38].

In 2012, Jinek et al. and Gasiunas et al. re-purposed CRISPR/Cas9 adaptive immunity as a biotechnological tool in E. coli, which subsequently become a versatile genome editing tool from microorganisms to animals [39], [40], [41], [42], [43], [44], [45], [46]. The CRISPR/Cas9 system comprises a single guide RNA (gRNA) and a Cas9 nuclease. The gRNA consists of CRISPR RNA (crRNA) and transactivating CRISPR RNA (tracrRNA) incorporating a tetraloop [38,[47], [48], [49]]. crRNA contains an approximate 20-nucleotide (nt) guide spacer sequence that can be readily programmed to target any DNA sequence [43]. Jinek et al. showed that a truncated tracrRNA containing nucleotides 23 to 48 retains cleavage activity [39]. The complex comprising gRNA and Cas9 will bind to the target sequence (protospacer) that is complementary to the guide spacer sequence, and a double-strand break is introduced in the vicinity of the protospacer adjacent motif (PAM), which in the case of SpyCas9 is 5ʹ-NGG (Fig. 1a). PAM is essential for self/non-self-discrimination during host immune defense [50]. The initial 7- to 12-nt sequence at the proximal end of the PAM is referred to as the seed region, which determines the affinity with which the Cas9–gRNA complex binds to DNA [51], [52], [53].

Following CRISPR/Cas9-mediated double-strand cleavage, the break is repaired via nonhomologous end-joining (NHEJ) or homology-directed repair (HDR) [54,55] (Fig. 1a). In *E. coli,* which lacks the NHEJ pathway enzymes, double-strand breaks are repaired via alternative end-joining mediated by recBCD [56], homologous recombination mediated by recA or overexpression of λ Red enzymes derived from bacteriophage [57]. Jiang et al. have demonstrated the application of a two-plasmid-based CRISPR/Cas9 system for continuous genome editing in *E. coli* [18], in which the expression of Cas9 and gRNA is partitioned in two plasmids and the repair template for HDR is supplied as a separate DNA fragment or within the gRNA plasmid. Continual genome editing can be performed by curing the gRNA plasmid from positively edited cells and introducing a new gRNA plasmid along with its repair template into the cells for the next round of genome editing. Using the CRISPR/Cas9 system, recombination rates of up to 97 % have been reported. CRISPR/Cas9 has demonstrated numerous applications for introducing scarless gene deletions, insertions, substitutions, and point mutations [58], [59], [60], [61], [62]. Other studies on the CRISPR/Cas9 system have focused on addressing challenges such as reducing the generation of escapers [63], engineering asymmetric homology arms for recombination [40], and enhancing gRNA stability [64]. Interested readers can refer to the previous review articles by Dong et al. and Hashemi et al. on the applications of CRISPR/Cas9 in metabolite biosynthesis [65,66].

## 2 CRISPR/Cas9-mediated interference

Although recombination efficiency has been substantially enhanced using CRISPR/Cas9-mediated double-strand breakage, simultaneous knock-out or knock-in at multiple loci in *E. coli* remains a challenge, and a less damaging tool is required to regulate the expression of multiple genes. Inspired by the gene-silencing mechanism, a number of studies have demonstrated the utility of synthetic sRNAs in blocking translation by binding to the mRNA in diverse bacteria, thereby knocking down target genes [67,68]. This strategy facilitates the post-transcriptional regulation of gene expression, which contrasts with the CRISPR interference (CRISPRi) system, in which CRISPR/Cas9 is re-purposed to knockdown genes at the transcriptional level. CRISPRi uses catalytically inactive or dead Cas9 (dCas9) to interfere with gene transcription. Using this system, the nuclease function of Cas9 is inactivated by introducing two point mutations, D10A and H840A, into the two catalytic domains to construct dCas9 [34,39,[69], [70], [71]]. Having the same traits as the Cas9 enzyme, the dCas9 enzyme retains the binding capacity of the parent enzyme but lacks its DNA cleavage activity.

Similar to the CRISPR/Cas9 system, CRISPRi can be readily programmed and activated. However, instead of introducing a double-strand break, dCas9 binds allosterically to its target gene. As a consequence, the dCas9–gRNA complex physically blocks the progression of RNA polymerase, subsequently interfering with gene transcription initiation and elongation [72], [73], [74] (Fig. 1b). As with other gene-silencing technologies, CRISPRi has been used to probe essential genes, the deletion of which proves fatal to organisms [75], [76], [77], [78]. The tunability and orthogonality of this system have been demonstrated in a diverse range of organisms, including bacteria [75,79,80], yeasts [42,81], fungi [82,83], plants [81,84,85], and mammals [86,87]. Herein, we provide a concise review of the applications of the CRISPRi system in *E.coli,* focusing on strategies developed to achieve precise control of gene repression, recent applications of the CRISPRi system, and the challenges and future perspectives of CRISPRi-mediated engineering. Recent advances in CRISPRi for high-throughput screening platforms have been reviewed previously and are not covered in this review [79,80,86,88].

## 3 Strategies for achieving precise control of gene repression levels using CRISPRi

An important advantage of the CRISPRi technology is its ability to achieve graded levels of target gene repression, thereby facilitating fine-tuning of the transcriptional output. Several strategies have been developed to successfully manipulate gene repression using CRISPRi as a transcriptional regulation tool, including engineered gRNAs [89], [90], [91], [92], [93], [94], [95], promoter regulation [96], [97], [98] and multi-layered CRISPRi genetic circuits to address retroactivity [99].

### 3.1 CRISPRi with engineered gRNAs

When using the CRISPRi machinery, the affinity with which the dCas9–gRNA complex binds to the target DNA is an important factor in achieving gene repression. The affinity of binding is dictated by the gRNA, particularly the 20-nt spacer sequence encoded by crRNA [37,100,101]. In the first demonstration of CRISPRi, Qi et al. revealed certain preliminary design rules for gRNA in the context of gene repression, notably with respect to spacer length, target loci, and the number of mismatches in the spacer sequence [89]. With regards to spacer length, a minimal 12-nt spacer sequence length is required to achieve gene repression. In terms of the target loci, it was established that varying the targeting site (for example, promoter or the gene sequence) of the spacer sequence, can significantly regulate the level of gene repression by up to 300-fold. Moreover, mismatches in the 20-nt spacer sequence were found to alter the binding affinity of the dCas9–gRNA complex to the target DNA.

**Fig. 2:**
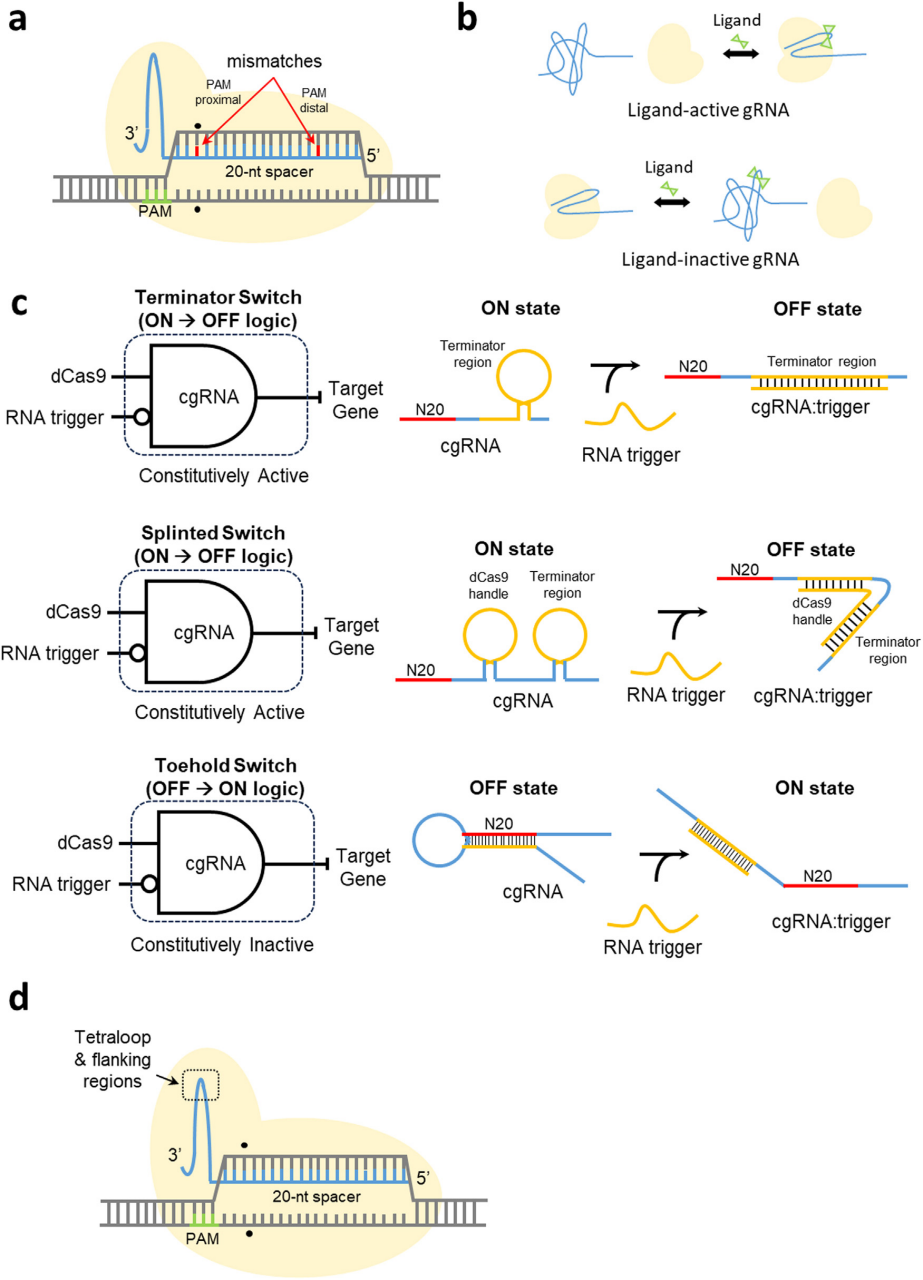
Strategies for the regulation of gene repression using engineered guide RNAs **a.** Strategic placement of nucleotide mismatches to alter binding affinity. **b.** Ligand-activated and ligand-deactivated engineered guide RNAs (gRNAs). **c.** Schematic diagram of Terminator Switch, Splinted Switch, and Toehold Switch AND-gates. For a Terminator Switch AND-gate, the gRNA contains an extended loop in the terminator region, which hybridizes with an RNA trigger to form a structure incompatible with the dCas9 protein (ON → OFF state). For a Splinted Switch AND-gate, the gRNA contains an extended loop in the dCas9 handle and terminator region, which hybridizes with an RNA trigger to form a structure incompatible with the dCas9 protein (ON → OFF state). For a Toehold Switch AND-gate, the target binding site of the gRNA is sequestered by a 5ʹ sequence to inhibit binding. The RNA trigger hybridizes with the 5ʹ sequence to de-sequester target binding, guiding the dCas9 to the target site (OFF → ON state). **d.** An engineered gRNA with modified tetraloop and flanking region mutations.

Among the aforementioned three strategies, recent studies have mainly focused on characterizing the binding affinity of the 20-nt spacer sequence with mismatches. By introducing mismatches, the affinity with which the dCas9–gRNA complex binds to the target DNA can be altered to regulate gene expression (Fig. 2a). As the binding affinity weakens, transcriptional repression relaxes, and in this regard, it has been demonstrated that strategic placement of mismatches in the PAM-proximal or -distal ends of the 20-nt spacer sequence can induce different degrees of relaxation in transcriptional repression. Vigouroux et al., who have studied the gene repression levels of various mismatches at the distal end of the PAM, observed reductions in the levels of gene expression with increasing spacer complementarity [90]. A model developed by Hawkins et al. to predict the activity of mismatched gRNA revealed similar patterns of gene expression, and it was found that the efficacy of the gRNA declines as the mismatch navigates closer toward the PAM-proximal end of the 20-nt spacer sequence [91]. Feng et al. subsequently performed a comprehensive landscape profiling of the gRNA mismatch effects [92], and consistent with previous studies [91], mismatches in the PAM-proximal region were found to be associated with a significant decline in binding affinity. In contrast, mismatches in the PAM-distal region were observed to coincide with a conservative reduction in binding affinity. Moreover, additive effects were also observed in the PAM-distal region in response to the introduction of multiple mismatches. The aforementioned observations present opportunities for quantitative customization of the binding affinity of the CRISPRi system. To intensify the distinction in gene repression, mutations can be introduced into the seed region to substantially weaken the binding affinity of the dCas9–gRNA complex to the target DNA. Moreover, to achieve a linear reduction in gene repression, mutation(s) can be introduced beyond the seed region and in the vicinity of the PAM-distal region.

Although varying degrees of gene repression can be achieved by altering the binding affinity of the dCas9–gRNA complex to the target DNA through strategic introduction of mismatches in the 20-nt spacer sequence, this approach increases the risk of reduced targeting specificity and increases in off-target effects. Previous studies have provided other structural insights into the gRNA that can be exploited for engineering [47]. In this regard, the repeat:anti-repeat duplex and stem loop 1 have been identified as key components facilitating the formation of a functional dCas9–gRNA complex [47]. In contrast, the linker and stem-loops 2 and 3 appear to be regions of lesser importance, although they provide further stability to the dCas9–gRNA complex [47]. These less essential regions tend to be more tolerable to mutations, thereby providing opportunities for engineering [47]. In this regard, Kundert et al. inserted an aptamer into the gRNA nexus to control the formation of the dCas9–gRNA complex via ligand binding [93] (Fig. 2b), and these authors also demonstrated that the design of the gRNA in a ligand-activated or ligand-inactivated state can achieve 11 × and 13 × dynamic ranges, respectively [93]. Furthermore, they obtained a near-linear, dose-dependent response of dCas9–gRNA complex activity by titrating the ligand concentration [93]. Simultaneous multi-gene repression has also been demonstrated using different ligands in an orthogonal and dose-dependent manner.

In a further study, Hanewich-Hollatz et al. demonstrated the use of conditionally engineered gRNAs to control the scope of gRNA activity [94]. Three conditionally engineered gRNAs, namely, the terminator, splinted, and toehold switches, were programmed at two levels, at which they were either activated (ON → OFF logic) or inactivated (OFF → ON logic) in the presence of an RNA trigger (Fig. 2c). There are two designs that follow the ON → OFF logic, the “terminator switch” and “splinted switch.” The terminator switch gRNA has an extended loop in the terminator region, whereas the splinted switch gRNA consists of loops in both the Cas9 handle and the terminator region. In both designs, an RNA trigger would hybridize with the gRNAs, thereby forming a structure incompatible with dCas9. In the reverse conditional OFF → ON logic, the target-binding region of the “toehold switch” gRNA is sequestered by a 5ʹ extension, thereby inhibiting binding. An RNA trigger would hybridize with the 5ʹ extension and de-sequester the target-binding region, thereby enabling the gRNA to guide the dCas9 to the target gene. Of the three aforementioned designs, the highest conditional response (15-fold) has been obtained using the terminator switch gRNA. However, although the conditionally engineered gRNAs are based on a two-state logic, these could potentially be used to cover a dynamic range of repression by titrating the amount of RNA trigger in a dose-dependent manner.

The aforementioned studies are effective to reduce the efficiency of repression but cannot increase it unless the expression of gRNAs is further enhanced. In this regard, however, Byun et al. have recently demonstrated that an increase in repression efficiency can be achieved without altering the expression of gRNA. Instead of incorporating additional elements into the CRISPRi system, Byun et al. constructed a library of engineered gRNAs with a tetraloop and flanking regions carrying mutations [95] (Fig. 2d). Using these engineered gRNA variants, reporter expression levels ranging from 8 % to 104 % were achieved, with certain designs being found to have higher repression efficiency than the wild-type sequence [95]. The findings of this study thus indicated that gene repression could be successfully tuned by engineering less essential regions of the gRNA. This accordingly provides the basis for an alternative approach that has noteworthy advantages over introducing mismatches in the 20-nt spacer sequence, namely, avoiding the consequences of losing binding specificity and a higher likelihood of off-target effects.

### 3.2 Promoter regulation

**Fig. 3:**
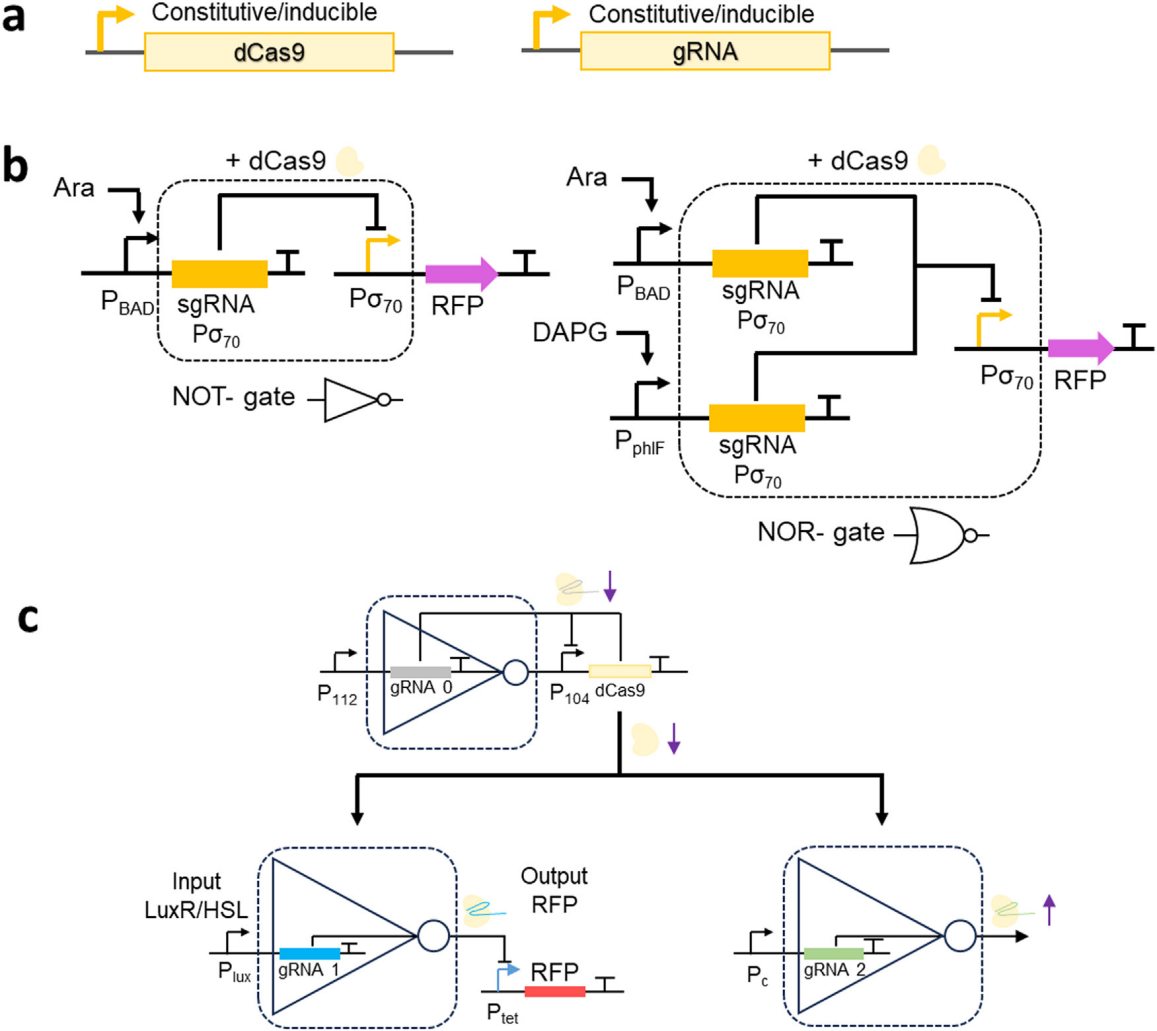
Strategies for regulating gene repression via promoter regulation and the use of multi-layered CRISPRi genetic circuits **a.** Titration of the CRISPRi machinery by promoter regulation. **b.** Schematic representation of the NOT-gate and NOR-gate. For the NOT-gate, the expression of gRNA Pσ_70_ is induced by arabinose to form a dCas9–gRNA complex that targets the σ_70_ promoter for the repression of a red fluorescent protein (RFP). For the NOR-gate, the expression of independent gRNA Pσ_70_ copies is induced by either or both arabinose and DAPG, thereby promoting the formation of a dCas9–gRNA complex that targets a single σ_70_ promoter for the repression of RFP. **c.** A CRISPRi module (CM) uses LuxR/HSL for the expression of gRNA 1 to form a dCas9–gRNA complex as an output that mediates the expression of RFP. The CM expresses a competitor, gRNA 2, that competes with the dCas9 protein. The available pool of dCas proteins is regulated by the dCas9 generator to neutralize the loading effect. The purple arrows indicate that in response to an increase in gRNA 2 to compete for more dCas9, the pool of dCas9 is reduced and this alleviates the repression of P104 to facilitate the production of larger amounts of dCas9 protein.

A further straightforward strategy that can be adopted to control transcriptional repression involves titration of the CRISPRi machinery. Several studies have accomplished this by incorporating inducible promoters in the CRISPRi machinery (Fig. 3a). By placing either dCas9 or sgRNA expression under the control of an inducible promoter, gene repression can be effectively fine-tuned in a dose-dependent manner. For example, Li et al. have developed a tunable CRISPRi (tCRIPSRi) system characterized by linear dose-dependent dCas9 expression under the control of the P_BAD_ promoter and expression of gRNA under the control of constitutive promoters [96]. Using this system, the quantitative control of dCas9 expression facilitated a more than 30-fold range in the repression of both essential and non-essential genes, with less than 10 % leaky expression. Similarly, by regulating the expression of gRNA under control of the P_Tet_ promoter, while constitutively expressing dCas9, Fontana et al. obtained gene repression ranging from 5- to 300-fold [97]. Moreover, these authors suggested that a 2.5-fold change in sgRNA expression could contribute to a 16-fold change in reporter gene expression [97].

Several studies have investigated the integration of Boolean logic operations, such as NOT- and NOR-gates, to construct CRISPRi genetic circuits for the regulation of gRNA expression [94,98]. Using this approach, promoter activity is input state-dependent, thereby facilitating precise output control. Nielsen et al. were the first to demonstrate the use of NOT- and NOR-logic gates in CRISPR/dCas9 circuits in *E. coli*, which were layered to construct a multi-input CRISPRi genetic circuit [98] (Fig. 3b). Furthermore, an orthogonal NOT gate was designed using a three-plasmid system for the expression of the red fluorescent protein (RFP), dCas9, and gRNA, respectively. Five synthetic σ_70_ output promoters were designed to control the expression of RFP, whereas the expression of dCas9 and gRNA are controlled by an anhydrotetracycline-inducible P_Tet_ promoter and an arabinose-inducible P_BAD_ promoter, respectively. Together, the dCas9–gRNA complex represses the σ70 output promoters of the RFP and can achieve high on-target repression of 56- to 440-fold. Similarly, a simple NOR-gate was constructed by connecting two input promoters, P_BAD_ and DAPG-inducible P_phlF_, for the transcription of independent copies of gRNA Pσ_70_ to repress a single σ_70_ output promoter. In the presence of both arabinose and a DAPG inducer, a 100-fold increase in repression was achieved from the σ_70_ output promoter.

### 3.3 Multi-layered CRISPRi genetic circuits

In the previous two sections, we have mainly described the various strategies adopted to fine-tune CRISPRi at the single-gene level. However, these strategies can potentially be applied to facilitate the regulation of multiple genes, thereby enhancing the scope and efficiency of transcriptional regulation. For example, transcriptional logic gates can be layered to construct multi-input genetic circuits that can be used to regulate multiple targets. Nevertheless, in the case of the multiplex CRISPRi machinery, the expression of any additional gRNA would sequester the shared dCas9 protein from other gRNAs. Accordingly, as the pool of gRNA increases, the availability of a finite pool of dCas9 protein becomes a bottleneck, a phenomenon referred to as retroactivity.

A potential solution to this obstacle lies in expressing the dCas9 protein at a higher level. However, this in turn, would lead to dCas9 toxicity [102,103]. To address this problem, Huang et al. constructed a dCas9 regulator that provides negative feedback on the levels of dCas9 using a CRISPRi Module (CM)-based NOT gate logic I/O response [99] (Fig. 3c). Specifically, they constructed two additional CMs, the first of which contains gRNA 1, which represses the transcription of the gene of interest, whereas the second contains gRNA 2, derived from the competitor CM, that competes for the same pool of dCas9 [99]. In the presence of both gRNAs, the pool of dCas9 is depleted. A dCas9 generator senses the reduction in dCas9 levels and de-represses the transcription of dCas9, thereby facilitating an increase in dCas9 transcription to compensate for the dCas9 load sequestered by gRNA 2. The dCas9 generator thus neutralizes the loading effect that arises in response to an increase in the pool of gRNA, while maintaining dCas9 expression at sufficiently low levels to prevent the cytotoxic effects of dCas9. The findings of this study accordingly indicate that multi-layer transcriptional logic gates could be used to address the problem of retroactivity associated with the addition of gRNAs. However, the number of gRNAs that can be co-expressed simultaneously at the highest dCas9 concentration without inducing retroactivity remains to be determined.

## 4 Applications of CRISPRi for metabolic engineering

Since the initial development of CRISPRi, in addition to the considerable efforts invested in designing a robust and regulatable CRISPRi system, the system has also been consistently employed for various applications. The most significant application of the CRISPRi system is the construction of microbial cell factories for the biosynthesis of valuable compounds via metabolic engineering (Table 1). In recent applications, CRISPRi has shown efficacy in probing essential genes [95,104,105], enhancing the availability of precursors [106], [107], [108], [109], reducing the diversion of carbon flux [110], and identifying positive gene candidates [111,112].

**Table 1:** Summary table of recent CRISPRi application in metabolic engineering.

Metabolites	Maximum Simultaneous target(s)	CRISPRi system	gRNA design	Fine- tuning	Final Gene target	Initial Yield	Final Yield/ Fold improvements	Reference
Lycopene	1	Singular	Individual gRNA expression cassette	Yes Engineered gRNA	gapA	–	2.7 fold	[95]
5-Aminolevulinic acid	1	Singular	Individual gRNA expression cassette	No	HemB	–	5.95 g/L (microaerobic) 6.93 g/L (anaerobic)	[104]
5-Aminolevulinic acid	1	Singular	Individual gRNA expression cassette	Yes Target loci	HemB	–	90.2 to 493.1 %	[105]
Phloroglucinol	1	Singular	Individual gRNA expression cassette	No	GltA	228 ± 15 mg/L	284 ± 8 mg/L	[108]
Aconitic acid	1	Singular	Individual gRNA expression cassette	No	icdA pykF	–	362.80 ± 22.05 mg/L; 60-fold (Shake flask) 623.80 ± 20.05 mg/L; 15-fold (fed-batch)	[110]
β-alanine	1	Singular	Individual gRNA expression cassette	No	ptsG	–	20 %	[111]
L-homoserine	1	Singular	Individual gRNA expression cassette	No	crr	–	5.52 g/L	[112]
Resveratrol	2	Multiplex	Multiple individual gRNA expression cassette	No	fabI	–	72 %	[127]
Dicinnamoylmethane	3	Multiplex	Multiple individual gRNA expression cassette	No	fabF fabD mdh	–	5.76-fold	[107]
Isoprenol	3	Multiplex	Multiple individual gRNA expression cassette	No	yggV accA	–	68.2 %	[131]
Cadaverine	4	Multiplex	Multiple individual gRNA expression cassette	No	ygjG	4 g/L	38 g/L	[132]
Malonyl-CoA/ (2S)-naringenin	5	Multiplex	Multiple individual gRNA expression cassette	Yes Promoter regulation	fabI fabH	–	29-fold 1073.8 mg/L (3 L bioreactor)	[106]

As mentioned earlier, CRISPRi enables the probing of essential genes that can potentially enhance the production of metabolites. For example, in the production of 5-aminolevulinic acid, 5-aminolevulinate dehydratase or *hemB* is an essential gene that catalyzes the conversion of 5-aminolevulinic acid to porphyrin [104,105]. To enhance the accumulation of 5-aminolevulinic acid, it is desirable to delete the *hemB* gene. However, given that *hemB* is an essential gene, its complete removal would impair cell growth. Miscevic et al. tackled this problem by using CRISPRi to reduce the carbon flux toward the biosynthesis of porphyrin by repressing HemB expression, thereby achieving 5-aminolevulinic acid titer of 5.95 g/L and 6.93 g/L under microaerobic and anaerobic conditions, respectively [104]. In a similar application of engineered gRNAs, Byun et al. also successfully repressed the expression of an essential gene, *gapA*, thereby achieving a 2.7-fold increase in lycopene production in the absence of growth defects [95].

A further application of CRISPRi is to obtain increases in the pools of biosynthetic pathway precursor molecules. For example, malonyl-CoA serves as an important precursor in the synthesis of numerous valuable chemical compounds, including polyphenols and fatty acids [106,107]. Wu et al. were first to demonstrate that an integrated CRISPRi system could be applied to tightly regulate the flux distribution of malonyl-CoA [106]. To reduce the utilization of malonyl-CoA in the fatty acid synthesis pathway, these authors used CRISPRi to simultaneously repress *fabI* and *fabH*, thereby achieving a 29-fold increase in malonyl-CoA levels. This increase in turn facilitated the production of (2S)-naringenin at titers of up to 1073.8 mg/L in 3-L bioreactors. Another important precursor molecule is acetyl-CoA, which leads to the synthesis of acetyl-chemicals and terpenoids. In a study designed to enhance phloroglucinol production from acetate, Yu et al. repressed the expression of endogenous citrate synthase (GltA) to reduce the competing carbon flux from acetyl-CoA to the TCA cycle, and hence increase the flux of acetyl-CoA toward phloroglucinol production [108]. By enhancing the carbon flux rewired toward phloroglucinol production, the titer of this product was raised from 228 ± 1 to 284 ± 8 mg/L [108].

Moreover, CRISPRi has also been applied to reduce carbon flux diversion. For example, Li et al. used CRISPRi to repress the activities of isocitrate dehydrogenase and pyruvate kinase, with the aim of diverting the carbon flux toward aconitic acid production [110]. By repressing the expression of *icdA* and *pykF* by 97.6 % and 99.2 %, respectively, they achieved respective reductions of 46.67 % and 50.25 % in the activities of isocitrate dehydrogenase and pyruvate kinase. This in turn contributed to 60- and 15-fold increases in aconitic acid production in shake flasks and fed-batch cultivation, respectively. In further studies, Ting and Ng used CRISPRi to enhance the production of cadaverine [109], in which four target genes, *speE, puuA, speG,* and *ygjG*, were selected to reduce the conversion of cadaverine to by-products. These four genes were examined through single and multiple gene repression, as a consequence of which, the repression of *ygjG* alone was found to be effective in significantly retarding the conversion of cadaverine to by-products, thereby raising cadaverine production by an additional 4 g/L to 38 g/L.

Regardless of metabolite, biosynthetic pathways are often confounded by the complex network of host metabolism and regulation. To enhance metabolite output, CRISPRi has been used to identify positive gene candidates among these complex networks [111,112]. For example, Liu et al. have used this approach to enhance the production of the amino acid l-homoserine, which serves as an important precursor for the synthesis of other valuable chemical compounds such as isobutanol [112]. To identify gene targets for modulation, they selected and repressed 39 genes associated with glycolysis, by-products, and branch pathways, among which 15 were subsequently shortlisted for individual gene deletion. The deletion of eight of these genes was found to contribute to a higher accumulation of l-homoserine compared with the control, with the highest titer of 5.52 g/L being obtained from the Δ*crr*-deleted strain. Similarly, Wang et al. studied the metabolic effects associated with the production of β-alanine by targeting 43 genes involved in either direct or indirect β-alanine biosynthetic pathways [111]. Estimated increases in β-alanine production of 20 % were achieved in response to the individual repression of *ptsG, ptsI, ptsH, crr, aroF*, and *aroG* gene expression. These promising gene targets were accordingly selected for deletion, and among the resultant deletion strains, the Δ*ptsG* strain was characterized by a more than 20 % enhancement in β-alanine production.

## 5 Challenges and future perspectives

Compared with CRISPR/Cas9-mediated gene deletion, the CRISPRi system has notable advantages that facilitate the manipulation of essential genes. Moreover, this system serves as a relatively simple screening platform that can be used to identify positive gene targets, and can be used to rewire metabolic fluxes in a graded manner. Nonetheless, there remain certain challenges that need to be overcome before the CRISPRi system can realize its full potential. Firstly, off-target effects remain an inherent problem of CRISPR systems [103]. These effects arise from the non-specific binding of the dCas9 protein or dCas9–gRNA complex to the target DNA [102,103]. Indeed, studies have shown that exceedingly stable target binding is established only with 9-nt PAM-proximal matches, suggesting that the chances of off-target binding are high [113,114] To address this issues, numerous *in silico* studies have yielded gRNAs designed to reduce the likelihood of off-target effects [66]. However, it is conceivable that some gRNA sequences might not offer the optimal repression efficiency of CRISPRi. For example, if the suggested target site were to be located toward the 3ʹ-end of the gene, the resulting repression efficiency would become lower than that obtained if the target site had been located near the 5ʹ-end of the gene [89]. In addition, the PAM requirement could be made more stringent to achieve higher targeting specificity [115,116]. Indeed, the use of Cas9 variants characterized by more complex PAM sequences, such as NmCas9, has been reported to be associated with fewer off-target DNA binding events, although such specificity may also limit the choices of suitable target site [117]. Engineered SpCas9 (eSpCas9) has also been demonstrated to have enhanced specificity and lower off-target DNA activity [118], and, similarly, an expanded PAM SpCas9 variant (xCas9) has been reported to have enhanced specificity with substantially lower off-target effects [119].

Secondly, the overexpression of Cas9 has notable toxic effects. Although catalytic inactivation of Cas9 prevents the nucleases from introducing cuts in the genome, the toxicity remains. In *E. coli*, the expression of dCas9 has been found to inhibit cell growth and induce development of an abnormal cell morphology [120]. To alleviate such toxicity, Zhang et al. constructed a dCas9 variant with a larger recognition region upon the removal of PAM binding [121]. With this modification, the *E. coli* cells could express 170-fold more dCas9 variant before the growth and cellular morphology were affected. The precise control of dCas9 expression can also contribute to alleviating cytotoxicity, as has been achieved using the dose-dependent control or genetic circuits described in sections 4.2 and 4.3. A further interesting strategy involves engineering smaller dCas9 molecules. In this regard, Shams et al. found that the dCas9 protein can tolerate deletions in its REC2, REC3, HNH, and RuvC domains while still retaining its function [122]. Shorter dCas9 sequences are advantageous in that plasmids can replicate more efficiently, and the expression of smaller proteins can reduce the burden incurred by resource demands, thereby reducing the toxicity to cells [123]. A further recent breakthrough in CRISPR-Cas technology has been the development of a miniature CRISPR-Cas system with SpCas12f mutants [124,125]. For example, Wang et al. recently demonstrated the use of the miniature CRISPR-AsCas12f1 system in *Bacillus anthracis* [125]. Given the nature of the smaller Cas protein, the size of the genome editing plasmid is correspondingly smaller, thereby facilitating higher transformation efficiency compared with Cas9 or Cas12. Such miniature CRISPR-Cas systems are likely to have further applications in the engineering of *E. coli*, which may contribute to alleviating the toxicity associated with the Cas protein.

Finally, given the challenges faced in achieving effective simultaneous multi-gene repression, the utility of multiplex CRISPRi systems in *E. coli* is still very much in its infancy. Although some studies have reported successful CRISPRi multiplexing, the application of these systems is still at the proof-of-concept stage, with at present up to five gene targets [126]. Moreover, reports indicate that a majority of multiplex CRISPRi applications are associated with growth defects or lower metabolite production when compared with single-gene repression or wild-type strain [107,109,127]. This could be associated with the toxicity of dCas9, which could be overcome by reducing or limiting the pool of dCas9 expressed. However, retroactivity or resource competition arises when the pool of dCas9 is not consistently available for the required application as the number of gRNAs increases. In multi-gene repression, the competition for resources is substantially magnified, and in addition to the examined pool of available dCas9, there may be other underlying limiting resources that have yet to be identified. For example, a limitation of essential shared resources, such as the RNA polymerase and ribosomes required for universal gene transcription and translation, may influence the performance of the CRISPRi system [128].

Given the on-going rapid advances in synthetic biology, metabolic modeling, and enzyme engineering, it is optimistic that the obstacles currently limiting the application of CRISPRi multiplex systems will gradually be overcome, thereby broaden their application for the repression of multiple genes in predictable and precise manners. Some strategies are applicable to emerging CRISPR/Cas9-mediated technologies such as base editing and prime editing [65,129,130]. Systems level approaches, such as transcriptomics and metabolomics, could provide further insights into the underlying mechanisms of CRISPRi. Armed with a better understanding of the mechanisms and the combined application of some of the strategies reviewed herein, we foresee further synergistic enhancements and more significant applications of CRIPSRi in the engineering of *E. coli* metabolism.

## CRediT authorship contribution statement

**Xiaohui Lim:** Writing – original draft, Visualization, Writing – review & editing. **Congqiang Zhang:** Writing – review & editing. **Xixian Chen:** Writing – original draft, Writing – review & editing.

## Declaration of Competing Interest

The authors declare that they have no known competing financial interests or personal relationships that could have appeared to influence the work reported in this paper.
